# Monitoring the mental well-being of caregivers during the Haiti-earthquake.

**DOI:** 10.1371/4fc33066f1947

**Published:** 2012-07-18

**Authors:** Marcel Van der Auwera, Michel Debacker, Ives Hubloue

## Abstract

Introduction During disaster relief, personnel’s safety is very important. Mental well being is a part of this safety issue. There is however a lack of objective mental well being monitoring tools, usable on scene, during disaster relief. This study covers the use of validated tools towards detection of psychological distress and monitoring of mental well being of disaster relief workers, during the Belgian First Aid and Support Team deployment after the Haiti earthquake in 2010. Methodology The study was conducted using a demographic questionnaire combined with validated measuring instruments: Belbin Team Role, Compassion Fatigue and Satisfaction Self-Test for Helpers, DMAT PsySTART, K6+ Self Report. A baseline measurement was performed before departure on mission, and measurements were repeated at day 1 and day 7 of the mission, at the end of mission, and 7 days, 30 days and 90 days post mission. Results 23 out of the 27 team members were included in the study. Using the Compassion Fatigue and Satisfaction Self-Test for Helpers as a monitoring tool, a stable condition was monitored in 7 participants, a dip in 5 participants, an arousal in 10 participants and a double pattern in 1 participant. Conclusions The study proved the ability to monitor mental well being and detect psychological distress, by self administered validated tools, during a real disaster relief mission. However for practical reasons some tools should be adapted to the specific use in the field. This study opens a whole new research area within the mental well being and monitoring field. Citation: Van der Auwera M, Debacker M, Hubloue I. Monitoring the mental well-being of caregivers during the Haiti-earthquake.. PLoS Currents Disasters. 2012 Jul 18

## Introduction

Regarding disaster relief, one of the most important issues for head of missions or relief directors is personnel’s safety. The hazards for the mental well-being of caregivers are part of this safety issue, and it isn’t new. Already in 1952 Killian reported symptoms of psychological discord, similar to the primary victims, in responders to a 1947 oil depot explosion^1^ . Disaster relief caregivers, often emergency or emergency-related responders, have to deal with the everyday stress of work, but also have the additional stress of being in a critical occupation^2^ . Furthermore serious operational tasks and physical demands are 2 of the 3 most severe stressors in emergency services personnel^3^ . Some disaster relief responders can handle this stress, others don’t^4^ . During disaster relief a form of self-neglect can be seen among relief workers, the feeling there is no time to rest can result in altered sleep patterns, and even more stress^5^ Also, responders to the same events are not all affected to the same degree, nor at the same time^6^ ^7^ . The importance of emotional preplanning is often underestimated and the research in the field of specific stress brought on by disaster relief needs gearing up^8^ ^9^ ^10^ ^11^ . Unspecific stress effects can be monitored with repeated self-tests comparing the results to a self-referenced baseline^12^ , also the additional stress of responding to disasters, should be monitored^13^ .

This paper covers the scientific study using validated tools towards detection of distress and monitoring of mental well-being conducted during the Haiti earthquake disaster relief deployment.

The hypothesis for this study being: “Every disaster relief-worker will during the harsh disaster-relief fieldwork, undergo a mental dip.”

## Methodology

The study being conducted during the Haiti earthquake disaster relief deployment, and the purpose of this scientific study being: using validated tools towards detection of distress and monitoring of mental well-being during disaster relief, made its methodology very concise.

**Population.** The study population was the personnel deployed as the second medical rotation off the Belgian First Aid and Support Team (B-FAST) on the Belgian field hospital after the Haiti earthquake disaster. This rapid response structure, with a view to sending emergency aid teams to a country or countries affected by a man-made or natural disaster, was created in November 2000 after earthquakes hit Turkey in August and November 1999. The objective is to give a structured, quicker and more efficient response to emergency situations.

The B-FAST Haiti 2 team was composed of 27 people. The team was composed of 1 head of mission, 6 medical doctors, 14 nurses, 1 pharmacist and 5 logistical members. The study inclusion was voluntary.

**Time definitions.** Baseline measurement was defined as the measurement just prior to departure, the measurement being before arriving on scene, it is will be annotated as Day 0 (D 0), if the travel to the disaster scene is short, or negatively as Day – x (D –x), x being the time needed to access the scene calculated in days .

Day 1 (D 1), being the first day of disaster relief by the team on site.

End of on scene work being the last day of field work, prior to returning home, will be will be annotated as Day x (D x), x being the on scene count of days being continued.

End of mission measurement was defined as the measurement just prior to be re-united with friends and family members at the return from mission; it will be annotated as Day x (D x), x being the on scene count of days being continued.

The 7 days post mission measurement was defined as the measurement 7 days after the homecoming; it will be annotated as Day x (D x), x being the on scene count of days being continued.

The 30 days post mission measurement was defined as the measurement 30 days after the homecoming; it will be annotated as Day x (D x), x being the on scene count of days being continued.

The 90 days post mission measurement was defined as the measurement 90 days after the homecoming; it will be annotated as Day x (D x), x being the on scene count of days being continued.

**Questionnaires.** The study was conducted using a demographic questionnaire combined with validated measuring-instruments: Belbin Team Role measurement, Compassion Fatigue and Satisfaction Self-Test for Helpers, DMAT PsySTART, K6+ Self Report measure.

*Belbin Team Role measurement*: Dr Meredith Belbin wanted to control the dynamics of teams to discover if, and how, problems could be pre-empted and avoided. His research at the Henley Management School in the UK started in the 1970’s. As research progressed, it revealed that the difference between success and failure for a team was not dependent on factors such as intellect, but more on behavior. The research team began to identify separate clusters of behavior, each of which formed distinct team contributions or “Team Roles”. Different individuals display different Team Roles to varying degrees. Reports identify Team Role preferences to allow an individual to appreciate where their strengths lie and which behaviors should be cultivated for the benefit of the team, as well as for individual development. A Belbin Team Role measurement attempts to measure an individual's behavioral contribution, through the lens of this theory. A theoretical approach, by David Straker, to well-balanced teams by using the Belbin Team Roles states that each team should have: a coordinator or shaper as leader, a plant to stimulate ideas, a monitor evaluator to maintain honesty and clarity and one or more implementer, resource investigator, completer-finisher to make things happen^14^ .

*Compassion Fatigue and Satisfaction Self-Test for Helpers*: The measure was originally called the Compassion Fatigue Self-Test and developed by Charles Figley in the late 1980s.In 1988 Beth Hudnall Stamm and Charles Figley began collaborating. In 1993, Stamm added the concept of compassion satisfaction and the name of the measure changed to the Compassion Fatigue and Satisfaction Test, of which the Self-Test for Helpers is one of the several versions^15^. Professional quality of life incorporates two aspects, the positive, Compassion Satisfaction (CS), and the negative, Compassion Fatigue (CF)^16^ . Compassion satisfaction is about the pleasure you derive from being able to do your work well. Compassion fatigue breaks into two parts. The first part concerns things such like exhaustion, frustration, anger and depression typical of burnout (BO). Most people have an intuitive idea of what burnout is. From the research perspective, burnout is associated with feelings of hopelessness and difficulties in dealing with work or in doing your job effectively. These negative feelings usually have a gradual onset. They can reflect the feeling that your efforts make no difference, or they can be associated with a very high workload or a non-supportive work environment. The second part of Compassion Fatigue, Secondary Traumatic Stress (STS) is a negative feeling driven by fear and work related trauma. Some trauma at work can be direct (primary) trauma. In other cases, work related trauma can be a combination of both primary and secondary trauma. Secondary Traumatic Stress is about work related, secondary exposure to people who have experienced extremely or traumatically stressful events. The negative effects of Secondary Traumatic Stress may include fear sleep difficulties, intrusive images, or avoiding reminders of the person’s traumatic experiences. Secondary Traumatic Stress is related to Vicarious Trauma as it shares many similar characteristics^17^ .

*DMAT PsySTART:* PsySTART is an evidence-based rapid mental health triage system and integrated disaster mental health incident management system. It has been developed by Merritt D. Schreiber at the Center for Public Health and Disasters, School of Public Health, UCLA Center for the Health Sciences. The DMAT version is the version for use during Disaster Medical Assistance Team (DMAT) deployment. The purpose of PsySTART is to identify responders who may be at risk for psychological stress reactions and may benefit from personal coping and/or screening from or referral to mental health support personnel. PsySTART measures only what has happened to an individual, based on what of stressful events personnel have been exposed to. PsySTART does not indicate overall mental health or mental symptoms, nor provides a diagnosis or formal assessment of mental health. The presence of any “yes” items is only an indicator that certain experience has occurred, not any single outcome. Individuals are, while checking the form, only confronted intellectually and visually with their own experienced events of that day. They can develop their own ways on how to use this information to support their own resilience and may elect to share it with mental health colleagues for additional coping ideas.

*K6+ Self Report:* The K6 measuring score is a shortened version of the Kessler Psychological Distress Scale-10 or K10. The K10 was developed in 1992 by Professors Ron Kessler and Dan Mroczek, as a short dimensional measure of non-specific psychological distress in the anxiety-depression spectrum, for use in the United States National Health Interview Survey. The K6 scale was introduced as a screening tool in the ‘redesigned’ US National Health Interview Survey. The K6 scale also screens nonspecific psychological distress, and is sensitive to DSM-IV^18^ mood or anxiety disorders. The K6 scale is shorter than the K10, 6 questions versus 10 questions, and is preferred because of its brevity and consistency across subsamples 19 20. The Self Report version is a self-administered version of the interviewer-administered one. The Self Report version also includes a number of other questions that are routinely administered to learn about persistence and impairment, the additional scoring is not required to score the K6 scale. There are 2 scoring systems available for the K6 scale: the original 0-4 scoring system, and the alternate Australian 1-5 scoring system. We use the 0-4 scoring system. The cut point for identifying a person at risk of psychological distress was set at 13.

The demographic questionnaire, the Belbin Team Role measurement and the Compassion Fatigue and Satisfaction Self-Test for Helpers were collected during the baseline measurement.

The Compassion Fatigue and Satisfaction Self-Test for Helpers was repeated at the end of Day 1, the end of Day 7, End of mission, 7 days post mission, 30 days post mission and 90 days post mission.

The DMAT PsySTART measurement was conducted every evening after on scene disaster relief, being from Day 1 trough End of on scene work.

The K6+ Self Report measure was collected 30 days post mission.

All data prior and during the mission was collected on paper, and manually transferred into a Microsoft® Office Excel spreadsheet. The data collected post mission was collected using an open source survey application, Limesurvey®, on the Faculty of Medicine and Pharmacy of the Vrije Universiteit Brussels server. The Microsoft® Office Excel spreadsheet was used for basic statistical and graphical processing.

**Team feedback and subjective interpretations.** After completion of the 90 days post mission measurement, all participants will receive a graphical view upon their own results. Included in the results file, every participant will also find a personalized interpretation of his results. The last part of the study being a qualitative feedback from the participants regarding there results and the interpretation of this results.

**Dip and arousal.** Within the interpretation and grouping off the results calculated in the Compassion Fatigue and Satisfaction Self-Test for Helpers measurement, we defined a dip and an arousal.

A dip being a drop in Compassion Satisfaction by more than 10% of the baseline value, and a simultaneous rise in Burnout and/or Secondary Traumatic Stress.

An arousal was defined as a rise in Compassion satisfaction by more than 10% of the baseline value, and a simultaneous drop in Burnout and/or Secondary Traumatic Stress.

**Informed consent and ethical approval.** The study was carried out as a pilot study in monitoring mental well being in disaster caregivers. Approval to the study was sought through the Federal Health Department advisory board of the Belgian First Aid and Support Team, B-FAST. An informed consent was sought from the participants on entry of the study, a second informed consent was sought from the participants on publication of the results.

## Results

The B-FAST Haiti 2 team was briefed concerning this study , informed consent and first questionnaires were handed out, on the airport prior to their departure.

**On scene key facts and figures.** The team worked 10 days on scene, triaged 3600 patients, treated 1200 emergencies in the field hospital and 1800 on out of hospital missions, performed 160 surgical interventions, assisted 3 deliveries and provided care for 20 hospitalized patients.

Every night there was a briefing by the head of mission, debriefing the day and planning the next day.

Almost half of the team faced a severe gastro-intestinal infection toward the end of the mission, several team members were put on IV-fluids.

A single intervention-critical incident stress debriefing was held 2 weeks post mission.

**Timing.** Travel to Haiti being difficult, the baseline data-collection was 2 days before arriving on scene, within the results the baseline will be annotated as Day – 2 (D -2).

The End of on scene work measurement was conducted on Day 10 (D 10).The End of mission measurement was conducted on Day 11 (D 11).The 7, 30 and 90 days post mission measurement were respectively conducted on Day 19 (D 19), Day 44 (D 44) and Day 104 (D 104).

**Population.** 23 out of the 27 team members were included in the study. 2 team-members did not want to participate. The minimum dataset for inclusion was: availability of the baseline Compassion Fatigue and Satisfaction Test measurement and at least 2 follow-up measurements. 1 participant did not meet the minimum dataset requirements and was excluded. The final team-member, being the study-investigator, was excluded on ground of bias.

Included were the head of mission, 6 medical doctors, 12 nurses, 1 pharmacist and 3 logistical members; being 18 men and 5 women. The mean age was 35.5 years, range 23 to 54 years. 5 included team members had previous mission-experience. 18 team members had direct patient contact during the Haiti mission, 5 did not.

**Belbin team role measurement.** Within the team we discovered: 9 team-workers, 7 implementers, 4 resource investigators, 2 shapers, 1 monitor evaluator and 1 coordinator.

The 2 highest scores, 55.4% and 51.4%, were measured in the team-worker role as primary role. All other team-members had a primary team role score lower than 40%, their scores were less pronounced and more spread over the different roles.

**Compasion, fatigue and satisfaction self-test for helpers.** Using the Compassion Fatigue and Satisfaction Self-Test for Helpers as a monitoring tool and using the ‘dip and arousal definition’ as a grouping tool, the following results were monitored.

A stable condition was monitored in 7 participants.

**Example 'stable condition':**
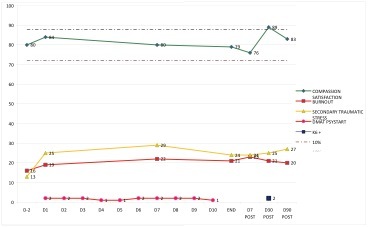

A dip was monitored in 5 participants. The dip started as a mean on D10 with a range from D4 till D18. The mean length is 56 days with a range from 34 to 97 days. The mean depth of the dip was a fall back in CS by 13.9%, with a range from 11.7% to 30.9%.

**Example 'dip':**
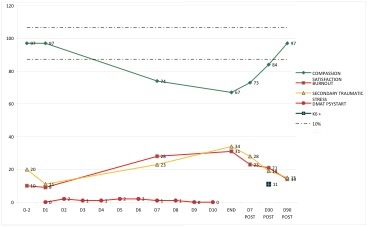

An arousal was monitored in 10 participants. The arousal started as a mean on D6 with a range from D1 to D73. The wide range suggests a pattern in starting day. The starting day of the arousal is situated within the first 2 weeks, 5 the first week and 3 the second week, or with a late onset, after D40. The mean length is 71 days with a range from 3 to 103 days. The mean peak of the arousal was a rise in CS by 18.6%, with a range from 11.3% to 26.4%.

**Example arousal:**
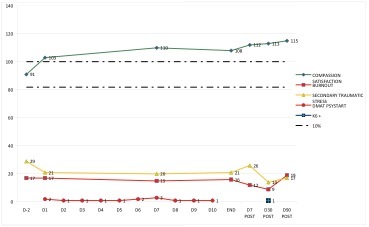

One participant had a double pattern within his monitoring. He started with an arousal on D9, which peaked at 27.1% and lasted for 6 days, and was followed by a dip on D17, which dipped at 21.4% and lasted for 87 days.

**Example 'double pattern':**
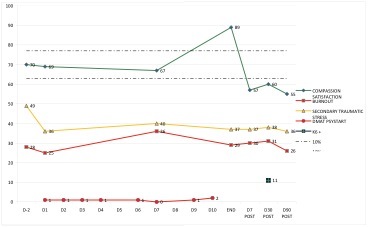

Crossing the dip and arousal data with the other collected data gave 3 significant correlations.

- Having no direct patient contact seemed to be more dip-inducing, p=0.016.
- Within the Belbin Team Roles, the participants with higher scores on the completer-finisher and the resource-investigator roles, were more prone to develop an arousal, p=0.026 and p=0.040.

**DMAT** **PsySTART measurement.** 206 DMAT PsySTART forms were collected during the 10 days of on-scene working.

13 participants entered all 10 forms. The number of forms per participant ranged from 5 to 10, the mean being 10.

The mean number of checked items on the form was 1, with a range from 0 to 6.

The total number of checked items during the mission, ranged from 0 to 30 with a mean at 12.

**K6 scale measurement.** 21 participants took the K6 scale measurement.

The median score was 2, with a range from 0 to 11. No participants scored above the cut point for psychological distress.

Overlooking the 30 days prior to the K6+ measurement, 4 participants reported being totally unable to work or carry out normal activities because of feelings of nervousness, hopelessness, restlessness or fidgety, depression or worthlessness, mentioned in the questionnaire. The median number of incapacity days being 4, with a range from 2 to 7. A doctor or other health professional was consulted by 2 of the 4 participants who reported incapacity. Physical problems, being the main cause of the mentioned feelings, were reported by 3 of the 4 participants.

**Team feedback and subjective interpretations.** After consulting their own results and the interpretation of these results, all participants agreed on revealing their results to the B-FAST management. One did ask to reveal the results in an anonymous file.

No real negative feedback was received, not regarding the study, not regarding the results or the interpretation of the results. The only criticism was towards the length of the Compassion Fatigue And Satisfaction Self-Test For Helpers questionnaire.******

The participants mostly agreed with the given interpretation of their results.

During the qualitative feedback the following statements were recorded from the participants: "wow, confronting, but instructive", "I think, speaking or myself, I experienced what the study shows", "Beautiful display of my inner self, during and after the mission", "I think it's true; I have absolutely no PTSS problems", "How annoying taking the test seemed, and how little faith I have or had in psychological questionnaires. I got a crystal clear view upon myself.", "I find myself in your analysis" and "Can I use this graph to prove my distress to my therapist?".

## Discussion

Conducting a prospective study, and its data collection, during on scene disaster relief isn’t an everyday experience. The purpose of this study being focused on the mental well-being of relief workers, made it possible. We tried to evaluate the use of validated tools towards monitoring of the well-being and detection of distress. The study proved the ability to monitor mental well-being and detect psychological distress, by self-administered validated tools, during a real disaster relief mission.

**Belbin team role measurement.** In our study none of the participants had a pronounced team role profile, except 2 participants, who had a team-worker profile as primary team role. The by David Straker[i]^21^ cited “theoretical requirements for a well balanced team” were met: our team had :

- A Shaper as medical team leader
- A plant to stimulate ideas
- A Monitor/evaluator to maintain honesty and clarity
- One or more Implementer, Team worker, Resource investigator or Completer/finisher to make things happen

The team role did not interact significantly with any other measured data.

**Compasion, fatigue and satisfaction self-test for helpers.** This study predefined 2 new interpretative tools: the dip an the arousal.

Monitoring the participants showed:

- 8 stable conditions
- 10 arousals
- 5 dips
- 1 double pattern: first arousal, followed by a dip.

Retrospective interpretation and feedback from the team revealed an overall recognition of the personal pattern within the subjective evaluation of the feelings during and regarding the mission.

The team members with an arousal couldn’t add any items in the feedback: they felt good doing what they did during the mission, the mission had been very stimulating and they were very pleased looking back to the experience. They did not feel the need to search for an explanation on how or why they felt well during the mission.

Interpretation from the dips revealed mostly an underlying feeling of distress: not being able to help as good as they would have imagined, not being able to deliver a good standard of healthcare, not being able to deliver other sorts of help (sheltering, food, …). The worst dip was detected in a logistical member, who had no direct contact with patients, and who felt guilty not being able to help these people.

Retrospective interpretation from the double patterned monitoring suggested: the pivot was induced by the participants’ physical problems. The team member was confronted with a gastro-intestinal infection, with its first manifestation of symptoms on D11, and which lasted for about 3 months, quiet similar to the monitored timing in the dip.

**DMAT PsySTART.** One participant, he entered 5 forms, did not check any event on his forms. During the feedback and interpretations he was confronted with this fact. He stated not having felt the urge to score the items. Surely he was confronted with very disturbing scenes, but the scenes were within the imagined ‘boundaries’. His coping mechanisms were already activated, just by reading the PsySTART form.

**K6 scale.** The highest score in our study was 11, with the cut point for identifying a person at risk of psychological distress was set at 13. Participant 22, who also had the worst monitored dip in the Compassion Fatigue and Satisfaction Self-Test for Helpers, had scored the 11. He did not pass the theoretical cut point, but he did seek mental counseling, and he used his measurements and feedback during the counseling.

**Critical incident stress debriefing.** After every deployment the Belgian First Aid and Support Team organizes a critical incident stress debriefing, CISD. This type of single session intervention was widely used with the aim of preventing continuing psychological difficulties in the past. However, recent studies and review showed there was a trend for increased self-report of post-traumatic stress disorder symptoms at 3 to 6 months follow-up in those who received this type of intervention[i]^22^ . Subjectively, all the individuals who participated in the CISD during this study reported it as being a very useful intervention. But the informal getting together after the CISD was reported even more pleasing and useful. This issue should be subject of discussion within the strategic planning of psychosocial monitoring of the disaster caregivers. An objective monitoring tool concerning mental well-being was however never used before, due to its unavailability.

**Limitations.** The small sample size, 23 participants out of a population of 27, will be the major limitation in this study. A 95% confidence interval on any statement concerning a binary question, yes-no for example, would be 29% large. The results of this study can therefore not be used for significant correlation statement between data.

The study period has been limited to 3 months post exposure. For monitoring and detection of distress purposes this period seems long enough. For post-traumatic stress follow up this period is much too short^23^ ^24^ , this however was not the aim of the study. Another aspect within this limitation is the fact that several participants did not regain their baseline level of CS during the study; the further evolution cannot be evaluated.

Another limitation within this study can be the use of a self-report methodology. Traditional criticisms of this methodology are response distortions and method variance. However, when taken into account in the construction of the questionnaires, and the analysis and interpretation of data derived from them, this limitation can be overcome. And even more: the self-report offers both practical and conceptual advantages to researchers in the social and behavioral sciences in general^25^ .

## Conclusions

The assumption made in the study modeling: “Every disaster relief-worker will during the harsh disaster-relief fieldwork, undergo a mental dip.”, has been rejected. Although the number of participants is too small to state significant conclusions, it is clear that not every relief worker undergoes a mental dip, several experience an arousal, and others are quiet stable in their mental well-being.

Regarding the above mentioned limitation, sample-size, we withhold ourselves of producing any statement regarding correlations, significances, sensitivity and specificity.

The study used validated tools in a new way: monitoring the mental well-being over a period of time by self-administered tests. These tools proved their ability to accomplish this monitoring. The participants, regardless of dip, stability or arousal, all subjectively, could agree with the monitored mental well-being levels and the interpretation this monitoring induced regarding their mission-experience.

This study method and monitoring tool could be of great use helping to identify prototypical patterns or trajectories of trauma reaction that include chronic dysfunction, but also delayed reactions, recovery, and psychological resilience.

The mental well-being and psychological support of relief workers can be seen as part of the employer’s duty and responsibility^26^ . Therefore, the continued use of the in this study developed tools can only be advised to any relief worker employer.

The main limitation in this study being the sample-size, we would recommend to continue using this methodology in larger studies, and evaluate the evolution of the mental well-being of relief workers in correlation to other factors.

In view of the time-limitation and the non-regaining of the baseline levels in CS, a prolongation of the study-period would be interesting, in order to evaluate the whole process over a longer timeframe in trajectories in people psychological responses.

The only criticism, from the participants, during the study was regarding the length of the Compassion Fatigue and Satisfaction Self-Test for Helpers: 66 questions. In future studies this questionnaire should be replaced by a shorter one.

The DMAT PsySTART has been designed as a rapid self-administered mental health triage system. When we start using it on a daily basis, a new redesigned card for use over several days would come in handy, rather than using a new card every day.

As a final conclusion we would emphasize that this study is only the starting point for a whole new research area within the mental well-being field.
